# The Sounds of Silence: Making Sense of the Absence of Domestic Violence Victims Help Seeking During the COVID-19 Pandemic

**DOI:** 10.1177/10778012241270267

**Published:** 2024-08-07

**Authors:** Lotta Agevall Gross, Johanna Thulin, Verner Denvall, Cecilia Kjellgren, Mikael Skillmark

**Affiliations:** 1 Department of social work, 8180 Linnaeus University, Växjö, Sweden; 2 8180 Linnaeus University, Växjö, Sweden; 3 School of social work, Lund university, Lund, Sweden; 4 8180 Linnaeus University, Växjö, Sweden; 5 4161 Jönköping University, Jönköping, Sweden

**Keywords:** domestic violence, COVID-19, social welfare, women's shelter, relational theory of risk

## Abstract

Increased concern was raised globally at the outbreak of COVID-19 that victims of domestic violence would be even more at risk when isolated with a violent partner and out of reach of support due to restrictions. Swedish staff in violence against women services prepared for increased calls for help. Instead, a worrying silence arose in a time and place of high uncertainty. This article analyzes the narratology of risk, when staff members in violence against women services, reflect upon their accounts, responses, and experiences, during the pandemic. The analysis is based on three themes, *accounting for expected increased influx, making sense of silence* and *accounting for mobilization*. The findings are discussed by applying the relational theory of risk.

When the COVID-19 pandemic broke out in the beginning of 2020 countries imposed restrictions to reduce the spread of infection. Restrictions in terms of social distancing implied measures like staying at home, working from home, and avoiding public areas. Social distancing also brings social isolation, which means that women exposed to interpersonal violence spend more time together with their perpetrators, which in turn increases the risk for violence (Myhill & Hohl, 2019; United Nations Population Fond, 2020). Workplaces and social networks are important respites for abused women, enabling the women to get some distance from their violent partner and facilitating the opportunity to seek help and leave the relationship (Agevall, 2012). Restrictions on women's mobility outside home might implicate that women exposed to domestic violence (continuing abbreviated DV) can’t receive support (Jarnecke & Flanagan, 2020; Kaukinen, 2020). The current study explores DV workers’ experiences of giving support to women exposed to violence during the COVID-19 pandemic with a special focus placed on how relationships of risk are established. The context of the study is distinguished by the fact that the DV workers had to navigate between two pandemics at the same time, partly the COVID-19 pandemic and partly the “shadow pandemic,” meaning DV.

Early in the pandemic, concerns were raised about how the imposed restrictions could affect the living conditions for women and children living with a violent partner. NGOs and professionals highlighted the risks and the government in Sweden responded by allocating extra resources to authorities working with DV. The concept of “shadow pandemic” was used to describe the violence that took place within the home during the COVID-19 pandemic. A parallel pandemic that accommodated hidden and underreported violence (UN Women, 2020a). The media also drew attention to several cases of severe violence during this time and played a crucial part in narrating the risks. Social workers and volunteers working with DV victims were forced to manage and balance the risks of two pandemics at the same time (Pfitzner, Fitz-Gibbon, & True, 2022; Pfitzner, Fitz-Gibbon, & Meyer, 2022; Skillmark et al., 2023). They became the frontline managing the “pandemic paradox,” balancing the risk of not staying at home due to COVID-19 with the risks of staying at home due to a violent partner (Bradbury-Jones & Isham, 2020; Burd et al., 2022). Social workers also balance the need to protect themselves, their colleagues, and clients from infection, and the need to provide quality support to their clients (Pfitzner et al., 2022a; Skillmark et al., 2023). Existing research evidence suggests that the new circumstances significantly affected the work situation of social workers (Banks et al., 2020; Kingstone et al., 2022). Care and support were now provided remotely, at a physical distance, and the private homes became the social workers' workplaces, which involved negotiating home/work boundaries (Kingstone et al., 2022; Pfitzner, Fitz-Gibbon, & Meyer, 2022). In managing both pandemics, professionals had to deal with the massive amount of information and disinformation that abounded during this time (Baines & Elliott, 2020; Ricknell, 2021). This infodemic was characterized by a high degree of uncertainty and sometimes conflicting information (Ricknell, 2021). There was uncertainty about how DV victims were affected by the COVID-19 pandemic and the imposed restrictions.

Uncertainty is inevitable in all social service work (Munro, 1996; Ponnert, 2013; Webb, 2006). This is partly because humans are unpredictable and cannot be easily read or controlled. Our lives are changeable and the problems that apply to one person cannot be easily translated to another. This is a form of uncertainty that all professionals need to deal with. Uncertainty concerns a lack of information in a changing world, weak follow-up systems and complexity in human service organizations that do not provide reliable knowledge (Hasenfeld, 2010). A central part of social work (SW) practice consists of responding to uncertainty, trying to make sense of and acting in relation to situations that are potentially harmful for individuals and families (Dominelli, 2002; Gregory & Holloway, 2005; Webb, 2006).

During the pandemic, this uncertainty became even more marked as professionals did not meet the women they feared were stuck with predators, and like many citizens around the world, received contradictory, varying information difficult to interpret. The silence from abused women was a real and worrying problem for front-line staff in violence against women services even if uncertainty is a common feature of their professional life. Professionals used different narratives to make sense of the absence of help-seeking women (Øverlien, 2020; Petersson & Hansson, 2022).

When COVID-19 broke out, several risk discourses arose and were active at the same time, such as, medical discourses, economic discourses, social discourses, and media discourses. Medical risk discourses played a crucial role in informing public health policies, creating clinical guidelines, and formulating mandates of individual behaviors in order to reduce the spread of the virus and minimize its impact on population health. The economic consequences as a result of COVID-19 were described as dramatic (Andersen et al., 2022; Neto & da Silva, 2023). In the media, there was constantly new information circulating about the effects of COVID-19 on societal institutions and its consequences for people (Ricknell, 2021). Parallel to these discourses, several social discourses were active with the spotlight directed toward various forms of vulnerability such as social isolation, mental health issues, increased economic vulnerability and alcohol consumption, and increased risk of exposure to violence (Akel et al., 2022; Morgan & Boxall, 2020; Pfitzner et al., 2022a; UN Women, 2020a, 2020b). In the article, we highlight the importance of understanding how discourses influence our understanding and management of risks, but also how different risk discourses are related to each other. How risks are presented and understood by different actors varies and affects the actions taken. If and how risks are identified thus becomes important for how they are subsequently managed.

When the social workers and volunteers try to navigate between these pandemics, within an organizational landscape surrounded by uncertainty, they need to rewrite the map, calculate and sketch out probability scenarios, anticipate the consequences of various interventions or lack of interventions, and act accordingly. It is thus the narratology of risk that falls under the scope of this study's research focus. In the qualitative interviews conducted within the current study, we get to follow professionals and volunteers working with DV victims when they reflect upon the situations of uncertainty that they had to relate to during the period of COVID-19 pandemic. The narratives told are social explanations and provide knowledge about if, why, and how something is considered a risk and what measures consequently ought to be taken. Risk is considered a discursive construction that shapes SW knowledge, practice, and ethics (Stanford, 2010). Risk is hereby studied as a social phenomenon, rather than risk per se. We join the theoretical tradition where perceptions of risk are seen as culturally biased by socially embedded values and beliefs. This means that risks are considered socially constructed, and vary with social, cultural, and historical contexts (Boholm & Corvellec, 2011; Douglas, 1992). In the current study, we want to understand how social workers and volunteers working with DV victims, conceive of and navigate between the two risks, the shadow pandemic and COVID-19. Drawing on Boholm and Corvellec's relational theory of risk, we explore how relationships of risk is established, that is, by studying how the risk object is linked to the object at risk (Boholm & Corvellec, 2011).

Previous research shows that staff in violence against women services expressed concerns when calls from women initially decreased and interpreted as women in isolation could not reach out for help. Further, when calls were received, staff experienced limitations due to restrictions to what extent they could support victims of violence. An Australian study from the early onset of the pandemic (June to August 2020) found that professionals working with abused women felt more pressure due to the pandemic (Carrington et al., 2020). They reported mixed experiences due to remote working where some reported difficulties concerning the technology, with a need for better equipment. Others found the transition smoother than expected and found it positive that their service had been modernized. However, there was a concern that some women were unable to contact them due to discomfort using technical equipment, lack of devices, or poor internet connection. The digital transition increased the work tasks including helping abused women adapt to the new technology and spending more time in trust-building. Adapting to new digital technology also required new risk assessments, to ensure that the perpetrator is kept outside the virtual room in which the victim receives support (Storer & Nyerges, 2023). Similar findings were reported by Moreira and Pinto da Costa who stressed how new technics demand professionals to learn how to use new devices and/or programs, but they also must adapt new strategies for securing the conversation with their clients (2020). The switch to digital meetings could form a barrier to seeking contact with professionals. Barriers could include uncertainty if the perpetrator could control digital devices or overhear the conversation but could also prohibit a disclosure when it wasńt an option to have a personal meeting (Moreira & Pinto da Costa, 2020; Pfitzner, Fitz-Gibbon, & True et al., 2022).

## A Relational Theory of Risk

According to the relational theory of risk, *risk is a product of situated cognition that establishes a causal and contingent relationship of risk between a risk object and an object at risk so that the risk object is considered, in some way and under certain circumstances, to threaten the value attached to the object at risk* (Boholm & Corvellec, 2011, p. 178). A relational theory of risk is based on the assumption that risk perceptions are formed by individual and collective understanding, continually reframed and redefined within a given social and historical context. It offers a theoretical framework to study how categories and classifications pertaining to risk are produced and reproduced, under conditions of uncertainty (Boholm & Corvellec, 2011).

For uncertainty to be transformed into risk, there needs to be a context in which something is perceived as a *risk object,* and an *object at risk,* and also a narrative that states a causational relationship between the two. Narratives based on, for example, test, statistics, and models are used to predict and assess probabilities in order to establish the correlation between risk objects and objects at risk (Boholm & Corvellec, 2011). According to Boholm and Cervellec, risk refers to uncertainty about and (the) severity of the consequences (or outcomes) of an activity with respect to something that humans value (Boholm & Corvellec, 2011, p. 177). A perception of risk is thus always normative. When the object at risk and risk objects are human beings, categorizations, and classifications will be central in the construction of value. In this article, the object at risk consists of DV victims. Accounts in relation to violence are never constant nor given but are shaped by conditions of knowledge production. The categories victim and perpetrator are concepts embedded in social and cultural beliefs. Social systems of shared values and beliefs decide who counts as an ideal victim worthy of support and protection from the society (Christie, 1986; Holstein & Miller, 1990). Depending on the narratology of risk, answers and responding acts will vary accordingly.

## The Swedish Context During COVID-19

There is a lack of sufficient Swedish data that provide an overview of men's violence against women in close relationships during the COVID-19 pandemic. To be able to determine an actual increase, a puzzle is needed, where several different sources need to be added and related to each other (Anderberg et al., 2022; Kaukinen, 2020). If we attempt to piece together how the situation developed in Sweden, we can see that crime in general is down signifcantly, both outdoor assault and indoor assault (Gerell et al., 2020). But upon closer examination, the number of reports concerning DV increases during periods when the restrictions and requirements to stay at home eased (BRÅ, 2022). A pattern that Sweden shares with other countries (Akel et al., 2022; Piquero et al., 2021). In 2020, the number of reported assaults against women increased by 4% in the first half of the year compared to the previous year (BRÅ, 2022). However, whether the increase is related to the pandemic is difficult to say, as the number of assaults against women has gradually increased over the years (BRÅ, 2022). Relying on crime statistics to create an understanding of the prevalence of violence is precarious though, as we only see the tip of the iceberg due to the large number of unreported cases. According to the National Security additional survey study, a different picture emerges (BRÅ, 2022). The results show an increase in the number of individuals subjected to various forms of violence, such as psychological, physical, material, digital, economic, and/or sexual violence, in Sweden from March to December 2020. More women than men were affected, and many of the affected individuals were in the younger age group of 16–24 years. The survey also found that the prevalence of victimization was highest among those who spent more time at home than usual during the pandemic. Among those who reported experiencing psychological violence, one-third felt that the violence had increased during the pandemic compared to before, while half of the respondents stated that they were subjected to it to the same extent as before the pandemic, and 13% said that the violence had decreased (BRÅ, 2022). Statistics on pathways to obtain help, such as the women's support line also indicates an increase in the number of women exposed to violence during the pandemic. Other sources reported an increase in the number of calls to womeńs shelters (WSs) during the pandemic (Unizon, 2024). COVID-19 affected all countries worldwide, but how it was handled in terms of restrictions and mandates varied. Many countries used a total lockdown in fighting COVID-19, but the Swedish Public Health Agency based their national strategy on recommendations, which, for example, urged citizens to test themselves for virus infection, stay home in case of symptoms, wearing facemasks, keeping distance, and avoid crowded areas. Except for restrictions regarding number of people in certain public areas or events, measures taken to reduce the spread of infection were mainly built on voluntary actions. It has thus been possible for people in Sweden to continue to visit their workplaces, grocery stores, and leave the children at daycare and schools if free from symptoms of infections. But even if there was no total lockdown, society was relatively closed. Many authorities, including social authorities, minimized the interactions with social service users and made adjustments to be able to reduce the spread of COVID-19 (Skillmark et al., 2023). Besides managing the regulations, and following new procedures in order to reduce the spread of COVID-19 disease, DV workers, still needed to continue their work, providing help and support to women who experienced violence from a violent partner. The organizational context for DV workers in Sweden can be either NGOs or social welfare.

The present study answers the call for more research concerning how DV agencies and shelters have developed strategies for meeting DV victims during the time of the pandemic (e.g., Ragavan et al., 2022).

## Method

To respond to the aim of the study, social welfare staff; social workers and managers, providing service for DV victims and volunteers and staff at WSs were interviewed on their experiences of serving victims of DV during COVID-19. The interviews were conducted in the second half of 2021. At that point, the pandemic had lasted for almost a year and a half, which enabled retrospective reflections over the time when information about the pandemic was disseminated and approaches to reduce the spread of infection had been implemented. The pandemic was an important political issue that was surrounded by various forms of national restrictions and recommendations. By that time over 70% of all individuals over 18 had received at least one dose of the vaccine and the restrictions gradually ceased during 2021 (Public Health Agency, 2024).

### Participants

The 16 participants, in the article referred to as DV workers, were all involved in supporting DV victims, within specialized units or functions, in the social welfare organization or a WS, run by a voluntary organization. Eleven social welfare employees, social workers or managers, employed in two municipalities in Sweden and five DV workers within WS in Sweden participated. Among the participants working within the social welfare organization, eight individuals had between 4 and 10 years of experience in working with intimate partner violence, while three individuals were relatively new to the field with approximately one year of professional experience in intimate partner violence. The five DV workers within WS had different educational backgrounds. One of the four employed had a university degree in behavioral science while the others had different educational backgrounds, like accounting, trade, finance, or commercials. They had all started by being engaged for years as volunteers at the WS prior to their current employment. They had then been employed at the shelter for between 3 and 17 years. The informants all had experience in working with the support and protection of victims of violence.

The specialized units had been established within the social welfare departments working with cases of DV. The two units served a population of about 170,000 people. Seven social workers were solely involved in the clinical work with DV victims, two team leaders (partly involved in clinical work with clients) as well as two managers participated in the study. Staff at the specialized units provides support, counseling, assessment, and placement in shelters for DV victims. The participating professionals were selected due to their roles as key actors in the daily task of organizing and providing support to DV victims in the organizations. One manager refrained from participating because of time constraints.

Among the participants working within the WS units, four were employed at a shelter. At one of the shelters, where only voluntarily engaged women assisted DV victims, a volunteer participated in the interview. The WSs are independent units, organizing and providing support to DV victims. Professionals as well as volunteers are involved with the support offered to women turning to the shelters. The staff/volunteers provide different forms of support to DV victims, as peer-support, counseling, and shelter. The WS units are affiliated with one of the two national organizations for WSs. Ten shelters were invited by mail to participate in this study and five responded they were interested in participating. The local area that the shelters served had populations from 17,000 to 140,000 inhabitants.

### Procedures

The individual interviews with social welfare staff were conducted using Zoom. The participants representing shelters were interviewed individually in face-to-face meetings. An interview guide was applied focusing on the DV workers' experiences of meeting victims of DV during the COVID-19 pandemic. Interviews lasted between 33 and 57 min. Author 5 and 3 conducted the interviews with social welfare staff and author 4 conducted the interviews with participants representing the shelters. The interviews were recorded and transcribed verbatim. A thematic-analytic approach was used to analyze the transcripts (Braun & Clarke, 2006, 2022).

### Analysis

The transcripts were read several times and the data were coded by authors independently. The authors categorized the texts into themes and categories. Finally, all authors discussed the content and categorization until a consensus was reached. Based on the theoretical framework, in which we explore the risk narratology of the two intertwined pandemics, the analysis led to a construct of three overlapping themes which structure the result of this study. The following themes were identified; *accounting for expected increased influx, making sense of silence* and *accounting for mobilization*.

Quotes from the interviews are presented indicating SW followed by number 1–11 for informants employed by social welfare and WS followed by number 1–5 for informants serving WS. The term informants is used interchangeably with respondents throughout the text.

### Ethical Approval

The study was approved by the Swedish Ethical Review Authority.

## Results

Based on the thematic analysis, three themes could be identified. The themes are presented time-chronological. The first theme *accounting for expected increased influx* relates to how the respondents initially made assumptions about how they expected the pandemic to affect their work. The second theme *making sense of silence*, reflects their understanding of the silence that arose. The third theme *accounting for mobilization,* focuses on the preparations and the informants’ descriptions of how they tried to manage the predictions that were made and prepare their work to meet the reality that was expected.

### Accounting for Expected Increased Influx

The first theme is a framing of the period when COVID-19 just broke out and mirrors the assumptions and expectations that respondents express on how the situation for DV victims would develop during the pandemic. This period is imbued by a high degree of uncertainty which affects DV workers in different ways. The first theme presented is thus about how uncertainty is managed and transformed into risk as a central part of the work done to meet the needs that were identified. An organizational context of relevance to understand how risk is interpreted and managed and how risk object is being linked to object at risk (Boholm & Corvellec, 2011).

Based on the scenarios presented by the national authorities and the media at the beginning of the pandemic, there was an assumption among the informants in social welfare and WSs that an increased number of victims of violence would turn to their agencies for support.

…we’re monitoring the news and I try to be prepared … and we believe it's highly likely that there's a risk of an increase in domestic violence as society becomes less open in general, but we haven’t yet seen the … the influx of clients we were expecting. There's been a slight increase now, but not at all in that way, so we’ve tried to look ahead /…/ that's kind of the idea, to try and be prepared. (SW 8)

The informants engaged in the WS services also expressed that they had the same concern about what the pandemic could bring in terms of consequences for women exposed to violence, like increased isolation. It was discussed both locally and with national organizations how to handle an upcoming increase in cases and how to support victims of violence while also taking into account the risk of the infection and the public health authority's recommendations.

The pandemic itself was very frustrating for us, because we went into it convinced that we’d need to be proactive. … Because there's going to be a lot happening now, when both partners are at home, we thought we’d see a huge need. (WS 1)

When the outcome was not as expected, with an increasing number of women who turned to the services for support, it was not understood by the informants as a sign of decreasing violence or reduced need for support. Rather, they maintained their belief that the violence had increased, and made assumptions about why the women were unable to contact them. The informants expressed thoughts that an increase in violence would become visible when the restrictions were eased, increasing the need for their service.

…as I said the other day, now that the restrictions have eased …. we have to be really aware of what's happening with us right now; whether the floodgates suddenly open or more people seek help, because it's been exactly like I and everyone else anticipated …. that more people are trapped, they are trapped with their abuser, they’re working from home, they can’t … like they’ve no opportunity, there's no respite like there is at work. (SW 2)

Similar to respondents at the social welfare organizations, respondents at WSs expressed a firm belief in their knowledge concerning risk factors for DV.

Quite early on we asked ourselves, what is this? You spend more time at home, and we were also thinking … we already know that in times of crisis and when people take time off and spend more time with their families it usually … there are studies that show that it increases, domestic violence… (WS 4)

All respondents expressed a strong belief that violence against women was increasing after the outbreak of COVID-19 and that the number of vulnerable women seeking help would rise. They relied on their knowledge concerning risk factors for DV, and their analysis of the situation following the pandemic (e.g., financial stress, more time spent together in the household) led to assumptions of higher risks for violence. Based on the assumptions of increased risk for women to be exposed to violence, they prepared themselves mentally to meet those help-seeking women expected to come.

### Making Sense of Silence

The second theme identified in the interviews concerns the perception of the silence the respondents experienced following the outbreak of COVID-19. Situations of uncertainty create a need for reason giving. Giving of reason is a social process, and is often about establishing, negotiating, and repairing social relations. Different social contexts require different forms of reason-giving. When the respondents give reason, they transform the public narratives that flourishes into specialized accounts, from formulas to cause-effect accounts, using what Tilly (2006) describes as technical accounts. This applies to the informants’ descriptions of the silence that arose when calls from women in need were not received at the expected level and how this could be understood. Instead of an increase in cases, most informants described that their centers were characterized by silence, fewer calls and visits. One employee at a WS expressed “And it's a bit … well it was quite surreal, it's a bit scary that it just suddenly went silent” WS 1.

The same experiences were recalled by the respondents working at social welfare:

Yes, how things worked … what struck me at the start of the pandemic was that the phones went quiet, it was …. And at the time I was working both with telephone counselling and investigations, and everything stopped …. it was like the phones stopped ringing, very few people got in touch at the start of the pandemic. (SW 6)

The respondents tried to understand what the silence was due to. Several described a continued belief that the pandemic increased vulnerability, but reduced the opportunities for victims of violence to seek help.

… so there was a huge decline during the fall, I don’t think we’ve ever had …. it was those who said, who’d been involved before, we didn’t have anyone at the refuge for a while, and I don’t think they’ve ever experienced that before. … And it was the same after Christmas, so you have to wonder whether those who needed help sought it earlier, or if they still haven’t managed to get help, that's the concern. (SW 1)

The opinion was shared with staff at the WSs. There was a big difference between the expected situation in relation to the silence that arose as few calls came from vulnerable women:

I know we all expected a lot of calls, but it didn’t turn out that way. I also think that women needed to be able to contact us at night … Maybe when he was asleep or out getting into trouble over something else, and then she would be able to call, that's my personal opinion. (WS 1)

Respondents expressed thoughts that abused women were more controlled and spent more time at home with their partners, which led to difficulties in making contact. As expressed by an employee at a WS: “When the husband is at home, can’t be at work or do their usual activities, her opportunities to spend time alone or leave the house are more limited, and it's harder to find opportunities to seek help” WS 2.

The respondents tried to be flexible and find ways to keep in touch with the women without their partners finding out about the contact. This could mean organizing the time for conversations when the woman was on special activities or making sure to be reachable at times that suited the woman.

We helped a woman … we had to time our calls to when she was driving, when she was sitting in the car …. That's when she could call, because it was the only time she had to herself …. On the way to nursery and on the school run, that's when we could talk to her and she said he's at home all the time …. So it's much, much harder to get in contact with us. So we definitely have cases like this where we’ve had to be flexible, to be available at a time that suits her. (WS 4)

This picture is shared by the informants within the social welfare units:

But what we’re seeing is that many … it's a bit difficult to reach those seeking help during a pandemic. … They’re at home all the time, many are at home with their abuser, the respite provided by going to work or school, being able to run an errand to coincide with that, or being able to call the helpline or go and see the school welfare officer to … just to talk and get help that way, it's all extremely limited. … There are many who don’t … we’ve found it hard to … to reach out to them, that they can … they can report something … they can find some way of contacting us for help, but then it's hard to maintain that contact. (SW 6)

One informant from social welfare describes the situation of two immigrant women who were victims of DV. They were affected when their introductory course in Swedish was shut down for a period, and their possibility of being in contact with social workers also was shut down.

Yes, it's … it's meant much greater isolation for them. … I’ve noticed that. I have two women that I can … who turned up now when you asked the question, who couldn’t get out to SFI [Swedish language classes for immigrants] for most of the pandemic and were isolated at home like that. Then when they eventually got out, when things started to open up that SFI … that they could go for lessons, these two women, independently of each other, got in touch and asked for protection … So this isolation has meant they’ve been unable … they can’t … get away as they did before, things were more intense at home, the violence escalated and they had no way of seeking protection. (SW 5)

According to the respondents, the predominant narrative around increasing violence in media may have contributed to fewer women seeking help. Women may shy away from seeking support to avoid being a nuisance. When the societal narrative claims that more people need support due to exposure to violence, it can mean that vulnerable women reduce their need for help in order not to be a burden for the helpers and society. As one employee at a WS says;

So I think lots of women … during times of uncertainty in society like we’ve had with the pandemic, many women don’t have the confidence to seek help, because they don’t know where … they hear that services are under pressure. … I think they play down their own issues; convince themselves that their problems aren’t that important. … It's like when you hear that the health services are overwhelmed and you feel that perhaps you don’t want to bother them with a sprained toe. … I don’t know, it's hard to say whether that's why because we’d been expecting…lots of people to contact us. … Instead it's been completely dead, which has meant I’ve had time to reflect on why, and I believe as women we’re just terrified of asking for help. … Not all, but many, and then maybe what will happen next, if I ask for help and there's no room at the refuge, what happens if I call but I don’t get an appointment. … They’re thinking the systems are overwhelmed and I can wait. (WS 3)

When the expected increase of cases didńt happen, respondents had to make accounts for the unexpected scenario—the silence. As parts of the society were shutting down, they describe telephones, chats and waiting rooms likewise turning quiet. However, they didńt expect the risk object DV becoming silent, rather they remained firm in their belief that there was an increased risk and consequently had to make accounts for why they couldńt reach the objects at risk.

One way to make sense of the silence was abused womeńs revalued victimhood. Considering oneself as a person “at risk,” in need of help and support, assumes a victim status, someone whós value is worth being prioritized in a time of crisis (Boholm & Corvellec, 2011; Christie, 1986). The COVID-19 pandemic challenges this process, when the government was admonishing people to stay at home, in order not to burden an already overloaded healthcare. The staff express their concern that women might therefore not seek the help that they need and have the right to receive.

Based on knowledge of the importance of alternative spaces for women's opportunities to question their relationship and seek support, new communication channels, increased accessibility and adaptations in the support activities are now being created so that they can offer support despite the pandemic and the restrictions that prevail.

The respondents are working with DV and are well familiar with the risk factors associated with DV. But the COVID-19 pandemic was a new phenomenon, Sweden has not before handled a pandemic (in modern times). Silence was also a narrative used in official reports concerning COVID-19 and DV in Sweden (Swedish Gender Equality Agency, 2021). No one knew what would happen, but staff in violence against women services transform their uncertainty into identifying risks—and making plans for managing them. In their narratives we can see individuals expressing a collective understanding of the risk object—violence against women will increase during the pandemic. That means they must find ways to act for helping the object at risk—the women exposed to violence—even when the women are not seeking help (see Boholm & Corvellec, 2011). The respondents try to explain the silence and try to understand where the abused women are and how they are doing. The same results from WSs can be seen in Petersson and Hansson's (2022) study. It is a reasoning to make the silence comprehensible. Silence as a theme can also be found in respondents’ stories in Øverlien's (2020) study from WSs in Norway. In a Swedish report from The Swedish Gender Equality Agency several professionals working with DV reports a silence. Some talk about a “huge silence” or a “frightening silence” (Swedish Gender Equality Agency, 2021, pp. 31–32). The report from the Swedish Gender Equality Agency highlighted increased risks for women who are exposed to violence due to COVID-19, but the report is not stating causality or establishing any connections (Swedish Gender Equality Agency, 2021). The guidance was thus scarce and the DV workers was navigating in a previously unseen landscape. A challenging job that was largely carried out from home, separated from colleagues and the professional framework that the social office entailed (Pfitzner, Fitz-Gibbon, & True, 2022; Skillmark et al., 2023). How the risks are to be understood is largely left to the professionals to manage. The respondents handled the new and unexpected scenario by relying on their previous knowledge concerning risks for DV. Their assumptions and conclusions lead to new ways of acting to reach the women.

### Accounting for Mobilization

From the beginning of the pandemic there has been an almost monotonous and unanimous voice warning for an increased influx of women experiencing DV. This has led to preparations and expectations for staff in violence against women services, both in Sweden and worldwide. Informants described a belief that DV will increase and continues to be preparing for the expected larger number of victims of violence seeking help. National and local authorities, as well as women's organizations, expanded their information channels to facilitate the victims in help-seeking. In the quote below we can see how the agencies designed different local strategies to reach out to potential victims.

…we’ve had campaigns; our municipal coordinator against violence in close relationships has … has been involved in running various campaigns on buses and supermarkets and other such places, where they’ve had … at the checkout they’ve had information about support and help for people suffering violence in close…. (SW 3)

The campaigns were massive and extensive in newspapers, television and advertisements, on a local as well as a national level. However, informants expressed doubts whether campaigns led to any evident increase in numbers of women contacting their service. One socialworker stated “…it was my colleague who was responsible for the campaign; I was informed about it of course, and we noticed a higher number of calls in connection with the campaign, but not as much as … as we were expecting” SW 7.

The respondents at the WSs describe how they tried to increase their availability, among other things by developing chat functions through which women exposed to violence could reach their service.

…we started up a chat function before the pandemic broke out, allowing people to contact each other that way, but it didn’t really take off … nothing happened and I … we run it together with our national organization, so I turned to them and asked what we were doing wrong, what else can we do…. (WS 1)

One WS that was more accessible and reachable around the clock was also the unit that described increased number of calls. Here, the respondents describe that the women probably found it more difficult to seek help during the pandemic because being forced to spend more time at home together with the man. The digital contact routes became a way to seek support and help when the opportunity arose. The largest increase in inflow of calls was described from a WS that had an emergency phone open 24 hr a day. The respondent from that site could describe that they received calls at night and calls from people residing in other parts of the country. Their emergency phone number was listed on the website of national association of WSs, which made it easily accessible to many people seeking help.

We’ve seen a huge increase during the pandemic … there have been many more, 100%, many more … both counselling on the phone, relatives who’ve contacted us, more women and children; we’ve also had men and nonbinary people getting in touch*.* (WS 2)

Informants from other WS that described an increased digital transition also experienced that the preparation and adaptation yielded results. One informant talks about their initiative for a local campaign and how they reached out with written information on different sites on how women could reach them.

And then we thought, we need … we have to come up with a different approach, how can we reach this target group? So we launched an information campaign quite early on, our communication team … Partly online, but then also … places we thought women and children might go, the pharmacy and shops, so we launched a massive information campaign quite early on … And launched the chat function, because it was … we realized that it might be difficult for people to call, so we put a lot of effort into marketing that. (WS 4)

How DV workers should meet abused women, digitally or physically, became a question for all informants, during the COVID-19 pandemic. Within social welfare, there were more central instructions on how the support work should be carried out during the pandemic. The informants at WSs described that the discretion for employees and volunteers was wide and that they felt free to decide how the contacts with victims of violence could be arranged.

… it came up in our discussions at a meeting, how we would react at the height of the pandemic, and we agreed that if you were unsure you didn’t have to travel and that it was ok to call someone else and ask if it was ok for them to travel, particularly at the critical stage when everyone was basically petrified of being physically close to another person … At that time you were a bit … many were reluctant to share their car with a woman they didn’t know, to sit with her and drive her from one place to another for perhaps half an hour … But I don’t think we encountered that situation, that we had to do anything differently, but we did discuss how we would handle it, because we had to do a risk assessment for our work … especially since we have a number of older volunteers, who we feel we need to take responsibility for. (WS 5)

All agencies implemented digital ways to meet with the women. However, there was resistance to excessively harsh demands for adaptations based on the pandemic. Professionals in one of the municipalities described the freedom to decide how they performed the work and there were social workers who preferred to meet the women on site over the digital solutions preferred by the organization. Even if the workplace had adopted restrictions the staff chose to follow more general guidelines of social distancing.

I thought we took a sensible approach and respected the situation, that we positioned the chairs as far away from each other as possible and that you could sit … our premises are small, we don’t have any large gatherings, in the waiting room we just had one client at a time. (SW 4)

In summary, the respondents describe extensive work to succeed in reaching out to vulnerable women while maintaining safety for helpers and victims of violence. They continued to withhold their view of the risk object increasing risks for the object at risk. The COVID-19 pandemic brought DV into focus, nationally as well as internationally. UN Secretary General Guterres as well as representatives of governments spoke of increased risk for DV and the need for knowledge and support. The increased focus, together with increased fundings could be seen as a positive scenario for DV workers. As respondents reveal they prepared themselves for meeting the increased need and delivering support—and were faced with a silence contradictory with what they as well as national and global leaders had pointed out. They couldńt use their competence, and feared they failed reaching the target group. Several different interventions were undertaken, in accordance with the risk assumptions made. The switch to remote work and use of digital solutions is primarily described as a solution, and something that helped the respondents continue their contacts or contact attempts (see also Skillmark et al., 2023). As in the study of Moreira and Pinto da Costa (2020) we could see how respondents tried to learn and adapt new strategies reaching their clients in a secure way.

### Discussion

By using a relational theory of risk we have shown that narratives of risk, were central to social workers' and volunteers' risk assessment and risk management, when navigating in between the shadow pandemic and COVID-19 pandemic. Establishing linkages between risk object and objects at risk was a consistent theme and decisive for the interventions. The COVID-19 situation was unique in many ways, but the uncertainty that is an inevitable part of SW remained.

In this paper, we have explored the narratives used when making sense of the silence that arose in the wake of COVID-19 and the absence of women that seek help due to exposure to DV. These narratives transformed uncertainty to risks, which enabled an understanding of the silence and absence of help-seeking women. Following the narratives, we see how respondents are trying to understand the risk object—DV and how it is affected by the risk object—COVID-19, in order to understand how it is affecting the object at risk—women experiencing DV. Basically, all narratives contain a feeling of uncertainty and even concern, but despite the lack of information about how the pandemic has affected the situation of women exposed to violence, none of the respondents express hesitating believes. Professionals in violence against women services are convinced that their assumption of an increased occurrence of violence remains. That is, their picture of the risk object is firm or even escalating. Their professional knowledge concerning the mechanisms of DV; the gender and power inequality, control, the limited room for action, increase of risk factors such as poor finances, unemployment, increased drug and alcohol consumption, lack of safe contexts and social interaction with others outside the relationship, are not challenged by the silence they are confronted with. It is rather the other way around, the silence is challenged through the risk narratives created by the professionals.

In a time of uncertainty, respondents relied on previous knowledge concerning risks, to make sense of the silence when navigating in the landscape of uncertainty that distinguished these two pandemics. To be able to mobilize for the situation right efforts, in a context of silence, the staff need to establish links between risk objects and objects at risk. Whether they managed to provide adequate support does not fall within the scope of this study, nor can we say something about the factual elements of the shadow pandemic in terms of numbers of victims. What we do want to highlight is the relevance of exploring how risk is constructed, interpreted, and handled, when depicting and analyzing SW practice. Risk management and risk government is not something given, but due to situated cognition, continually redefined, and reframed in a social and cultural context. In DV work uncertainty is ever present, and the transformations of uncertainty into risk is a part of SW practice, with implications for both professionals and social service users.

With this in mind, what can we learn from the risk management described in the article? We would like to underline the importance of professional discretion, when trying to understand and respond to identified risks. The neo-liberal risk society has shaped and reordered SW practices during recent decades, and shifted its focus from presented needs and resources to risks and security (Kemshall et al., 1997; Webb, 2006). Logics of regulation and security constituting the framework for understanding SW, cannot answer to the questions of silence. A one-sided focus on the visible, would not have explained the silence from women subjected to violence in their homes. The silence regarding DV is not unique to the pandemic; it exists nonetheless, considering the high levels of underreporting. What became a unique situation was that professionals now had to handle the fact that women were locked in with their abusers, in a society where pathways to support and assistance were limited. When narrating risks, in a landscape of silence, the social workers had to abandon a focus on the visual and measurable, and use a holistic perspective grounded in previous experiences and encounters with victims of DV. To address the silence, uncertainty, and constructed risks, it required, according to Banks et al. (2020) and other researchers, strong ethical principles and ethical logistics. The silence and the absent surge of people seeking help became an incentive to create new ways of contact and methods to provide support to women who were subjected to violence during the pandemic (Banks et al., 2020; Romakkaniemi et al., 2021; Webb, 2006).

### Limitations and Implications for Practice

Due to the size of the sample, generalizations should be made with caution. It is also important to keep in mind that the organization of DV support varies, not least when it comes to WSs in Sweden. On the other hand, case studies expand our understanding of individual phenomena and provide the opportunity for theory development (George & Bennett, 2005). Bearing this in mind, we believe that the results can be of importance when it comes to reflecting on uncertainty as an inevitable part of SW, especially in the field of DV. Human service organizations must strive for delivering trustworthy and comprehensible information to frontline professionals. Understanding risk factors and the relationship of risk is of high relevance for SW and lays the foundation for a proactive and effective risk management.
